# Primary Ovarian Insufficiency Induced by *Fanconi Anemia E* Mutation in a Mouse Model

**DOI:** 10.1371/journal.pone.0144285

**Published:** 2016-03-03

**Authors:** Chun Fu, Khurshida Begum, Paul A. Overbeek

**Affiliations:** 1 Department of Obstetrics and Gynecology, Second Xiangya Hospital, Central South University, Changsha, Hunan, China; 2 Department of Molecular and Cellular Biology, Baylor College of Medicine, Houston, Texas, United States of America; China Agricultural University, CHINA

## Abstract

In most cases of primary ovarian insufficiency (POI), the cause of the depletion of ovarian follicles is unknown. Fanconi anemia (FA) proteins are known to play important roles in follicular development. Using random insertional mutagenesis with a lentiviral transgene, we identified a family with reduced fertility in the homozygous transgenic mice. We identified the integration site and found that the lentivirus had integrated into intron 8 of the Fanconi E gene (*Fance*). By RT-PCR and in situ hybridization, we found that *Fance* transcript levels were significantly reduced. The *Fance* homozygous mutant mice were assayed for changes in ovarian development, follicle numbers and estrous cycle. Ovarian dysplasias and a severe lack of follicles were seen in the mutant mice. In addition, the estrous cycle was disrupted in adult females. Our results suggest that POI has been induced by the *Fance* mutation in this new mouse model.

## Introduction

Healthy ovarian function is vital for reproductive success and for general health in women. Primary ovarian insufficiency (POI) is a type of ovarian dysfunction that results in premature menopause, usually at less than 40 years of age [1, 2]. Clinical manifestations in patients with POI include primary or secondary amenorrhea, infertility, menopausal syndrome and increased risk of osteoporosis and cardiovascular disease [3]. Many factors may contribute to ovarian insufficiency, including genetic defects, autoimmunity, chemotherapy, and radiation therapy [4]. Although the cause of POI is undefined in most cases, the disorder is generally associated with the dysfunction and/or depletion of follicles [2, 5].

Some tests of ovarian reserve have been developed, such as measurement of hormone levels, follicle count by transvaginal ultrasound, and laparoscopic biopsy of the ovarian cortex [2]. The information from these tests is limited. Nonetheless, the clinical results show that normal ovarian function requires the presence of a sufficient number of primordial follicles. The intial pool of primordial oocytes is generated by rapid proliferation of primordial germ cells. Accurate repair of any DNA damage that occurs during this rapid proliferation is essential to protect against the accumulation of new mutations in the germ line. The Fanconi anemia (FA) genes encode a set of proteins that interact to mediate DNA damage repair. DNA damage at replication forks may activate the FA pathway in germ cells [6].

To date, 18 FA genes have been identified as mutated in human patients [7–9]. Mouse models have been generated for some of these genes. All of the FA mutantmouse models, such as Fanconi anemia group A (*Fanca*), -*c*,*-d1*,*-d2*,*-f*,*-g*,*-i*,*-m*,*-n*,*-o* and–*p*, have gonad dysplasia and impaired reproductive ability [10–20]. Ovaries of homozygous mutant mice show follicle depletion and interstitial cell hyperplasia, which is similar to POI patients. These findings suggest the FA genes play an important role in the regulation of germ cell development.

The Fanconi anemia E (FANCE) protein binds to the FA core complex, and is thought to form a bridge between the FA core complex and FANCD2 by directly binding to FANCC and FANCD2 [21, 22]. To date, there has been no *Fance* mutant animal model described. Hence, the roles of FANCE for follicular and ovarian development are unclear. We have generated a novel *Fance* mutant mouse line by random insertional mutagenesis. We describe herein the phenotype of the homozygous mutant females, which includes alterations in follicle development and compromised fertility. Our results show that the *Fance* mutant mice provide a new animal model of POI.

## Materials and Methods

### Generation of the *Fance* insertional mutation and mouse genotyping

All Animal experiments were performed using mice from an FVB/N background and were approved by the Institutional Animal Care and Use Committee at Baylor College of Medicine. Experimental mice were maintained under a 14 h light/10 h dark cycle at 21°C with food and water available ad libitum. All efforts were made to minimize the number of animals used and to minimize their suffering.

A self-inactivating lentivirus carrying a tyrosinase miningene was packaged and used to generate transgenic mice by injection under the zona pellucida of albino FVB/N two-cell stage mouse embryos. Potential founders were identified by inspection for pigmentation. Expression of the tyrosinase minigene results in melanin production and gene therapy for albinism. Founder mice were bred to albino FVB/N mice and F1 offspring with distinct coat colors (i.e., with different sites of integration in the genome) were used to establish transgenic lines. New lines were inbred to generate homozygous transgenic mice, and the homozygotes were assessed for altered phenotypes. For line OVE2364E-2a2, the homozygous mice showed reduced fertility. Using an inverse PCR protocol, the lentiviral integration site was amplified and sequenced (see S1 Fig). A BLAT homology search revealed that the lentivirus had integrated cleanly into intron 8 of *Fance* on chromosome 17. PCR primers were designed with homology to the genomic sequences upstream and downstream from the integration site (see below).

Genomic DNA was extracted from tail snips of 3-week-old mice. The tails were put into 160ul tail lysis buffer (containing Proteinase K 200ug/ul) and digested overnight @60°C, then heated @ 95°C for 30 min to inactivate Proteinase K. After centrifugation, PCR reactions were performed with 1 ul genomic DNA and 12.5 pmol each primer. *Fance* mutant mice were genotyped using primers LF (left flank) 5’-TGGCATCTCCACTTCTCTATCA and RF (right flank) 5’- AGAGCAGCCTGGACTACTTGAG and the following conditions: 94°C for 3 min; 35 cycles of 94°C for 30sec, 54°C for 50sec, and 72°Cfor 70sec; followed by a final extension step at 72°C for 5 min. PCR products were separated by electrophoresis on a 1% (wt/vol) agarose gel. Mice with a wild type copy of *Fance* intron 8 produce an amplification band of 620 bp, while the homozygous mutants show no amplification band (due to the presence of the intervening lentiviral sequences).

### RNA extraction, RT-PCR and real-time PCR

Mouse tissues (ovary, liver and lung) were harvested and homogenized by rotor-stator. Total RNA was extracted using the RNeasy Mini Kit (Cat# 74104, Qiagen). First-strand complementary DNA (cDNA) was synthesized using oligo(dT) primers and the SuperScript^®^III first-strand synthesis system (Cat# 18080–051, Invitrogen). The region of the Fance transcript from exon 6 to exon 10 was amplified using a sense primer (5’-cctcgtctccttctgtgtaaagt) and an antisense primer (5’-tgtgttctgagacaagcagtcag). The hypoxanthine phosphoribosyltransferase (Hprt) transcript was amplified using sense primer 5’-atgacctagatttgttttgtatacc and antisense primer 5’-gtagctcttcagtctgataaaatctac. The RT-PCR conditions were as follows: 94°Cfor 2 min; 35–39 cycles of 94°C for 30sec, 54°C for 50sec, and 72°C70sec; final extension step at 72°C for 5 min.

Real-time PCR amplifications were performed with the PerfeCTa® SYBR® Green SuperMix (Cat# 95055–100,Quanta BioSciences) and the StepOnePlus real-time PCR system (Applied Biosystems) as follows: 2min at 50°C,10min at 95°C and then 40 cycles of 15 sec at 95°C, 30 sec at 60°C and 45 sec at 72°C. Relative transcript levels were obtained by subtracting the threshold cycle (Ct) value of the housekeeping gene (*Hprt*) from the corresponding Ct value of *Fance* gene. Differences in mRNA levels were calculated according to the expressionΔΔCt, the relative target number then is 2^-ΔΔCt^ [23].

### Synthesis of *Fance* RNA probe and in situ hybridization(ISH)

Mouse *Fance* coding sequences from exon 2 to exon 10 were amplified by RT-PCR using primers 5’-ggatgtgtcctctaccactgatg-3’ (sense) and 5’-cctggatggactgtcttcttt-3’ (antisense). The PCR product was ligated into a TOPO vector (pCRTMII-TOPO vector, Cat# K4600-01, Invitrogen). Linearized plasmid was used for in vitro transcription to incorporate digoxygenin-labeled UTP (DIG RNA Labeling Kit, Cat#1175025910, Roche). Sense and antisense probes were generated using SP6 or T7 polymerase, respectively. In situ hybridizations were done in the In Situ Hybridization Core Lab of the Baylor College of Medicine using frozen sections from mouse ovaries at two different ages (2-week-old and 8-week-old, n = 2).

### Mouse phenotyping and mating

Matings of heterozygous transgenic mice produced wild type (*Fance*^+/+^), heterozygous (*Fance*^+/-^) and homozygous mutant (*Fance*^-/-^) offspring in a 1:2:1 ratio. Weights were recorded weekly. We found no differences between the wild-type *Fance*^+/+^ and the heterozygous *Fance*^+/-^ mice. So mice were divided into 2 groups to observe. One was a control group containing *Fance*^+/+^ and *Fance*^+/-^ mice, and the other group included *Fance*^-/-^mice. Each group had 8 mice. Reproductive organs were obtained from 1 week to 8 weeks of age.

For fertility testing, 4 to 6-week-old *Fance*^-/-^ and littermate control females (either *Fance*^+/+^ or *Fance*^+/-^) were paired with an FVB male mouse. Two female mice were paired with a single male mouse in one cage. Cages were monitored daily for 6months. The times of pregnancy, the numbers of litters and litter sizes were recorded for each mouse.

### Superovulation procedure

Female mice at4weeks of age were superovulated by intraperitoneal injection with 5U pregnant mare serum gonadotropin (PMSG, Intervet, Holland), followed by injection of 5 IU of human chorionic gonadotropin (HCG, Pregnyl, Organon Laboratories Ltd) 48 hr later. The mice were then paired with an individual male that had previously been tested for fertility. The following morning the mice were inspected for a vaginal plug. Female mice were sacrificed using controlled release of CO_2_, and one-cell stage embryos were isolated.

### Histological analyses of ovaries by hematoxylin and eosin (H&E) staining

Ovaries were stored in 4% paraformaldehyde overnight at 4°C, dehydrated and embedded in paraffin. Ovaries were serially sectioned at a thickness of 5μm. Every sixth section was stained with H&E for morphological observation. Based on the standards established by Ren [24], ovarian follicles at different developmental stages, including primordial, primary, secondary and antral follicles were counted in the stained sections of each ovary.

### TdT-mediated dUTP nick-end labeling (TUNEL) assay

Apoptosis in ovarian cells was determined with a TUNEL assay kit (Fluorescein Roche, 11684795910). TUNEL analysis was performed on paraffin-embedded ovaries according to manufacturer’s instructions. Sections were counterstained with DAPI (Vector Laboratories, H-1200). A positive control slide was prepared by incubation with DNaseI(Sigma, D-4527) for 30 min at room temperature. The negative control slide received 2ul of distilled water in place of 2ul of TdT2 in the fluorescein-12-dUTP reaction mixture.

### Assessment of the estrous cycle

Vaginal cytology was used to assess the estrous cycle at 3 different ages (9 weeks, 13 weeks and 20 weeks). Each group included 2 *Fance*^+/+^ mice and 4 *Fance*^-/-^ mice.

Vaginal exfoliated cell smears were prepared at 8AM for 28 consecutive days. The vaginal epithelium was washed with 100μl of autoclaved 0.9% NaCl and part of the washing solution was transferred onto a microscopic slide. After air drying, slides were stained with H&E. Cell morphology was microscopically evaluated and stages of the estrous cycle were determined according to the published literature [25]. All slides were analyzed by two independent evaluators. The cycle phases were defined as follows: proestrus, nucleated epithelial cells with occasional leukocytes and small cells with degenerative nuclei; estrus, numerous cells from squamous epithelium and small epithelial cells; metestrus, numerous leukocytes and small cornified epithelial cells; diestrus, mucus and nucleated cells.

### Statistics

The data are presented as mean ± standard deviation. The differences in relative transcript levels, body weights, weights of the reproductive system, number of pregnancies, average litter sizes, and the number of follicles were compared by independent sample t-test by SPSS software (version 17.0). The ratios of estrus phase and diestrus phase were compared by Fisher exact test. P value <0.05 was defined as statistically significant.

## Results

### 1. Genotype: phenotype analysis

**Fig 1:**
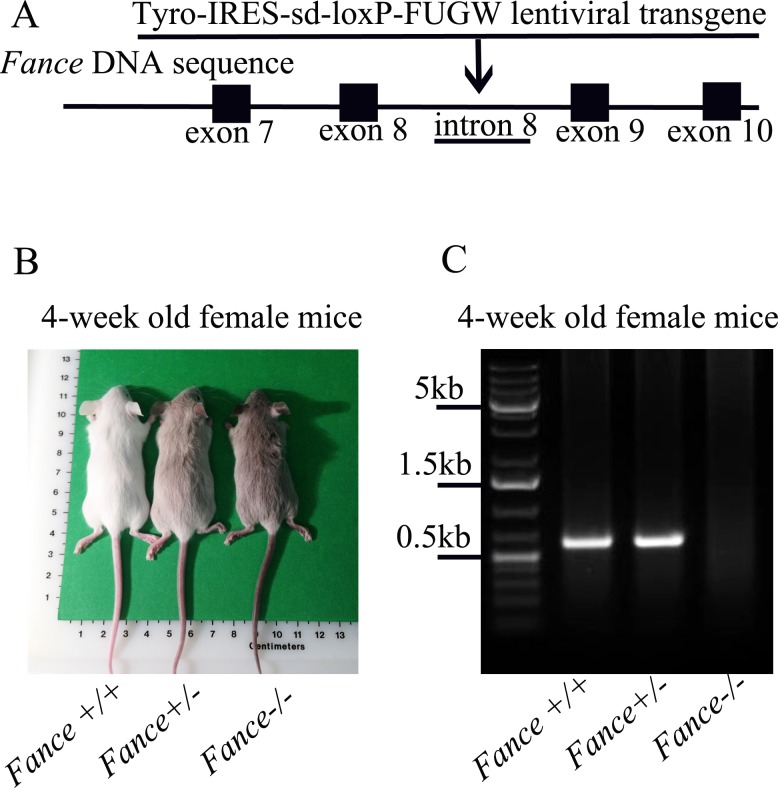
*Fance* insertional mutation. (A) Construct map of the Tyro-IRES-sd-loxP-FUGW lentiviral transgene, and schematic of the lentiviral integration site in intron 8 of Fance. (B) Coat color of female mice (4-week-old). (C) PCR genotyping of mouse tail DNA. Expression of the tyrosinase minigene (Tyro) in the transgene results in pigmentation in the transgenic mice. Mice with different genotypes show different coat colors: *Fance*^+/+^ (wild-type) are albino; *Fance*^+/-^ (heterozygotes) are light gray; *Fance*^-/-^ (homozygotes) are darker gray.

**Fig 2:**
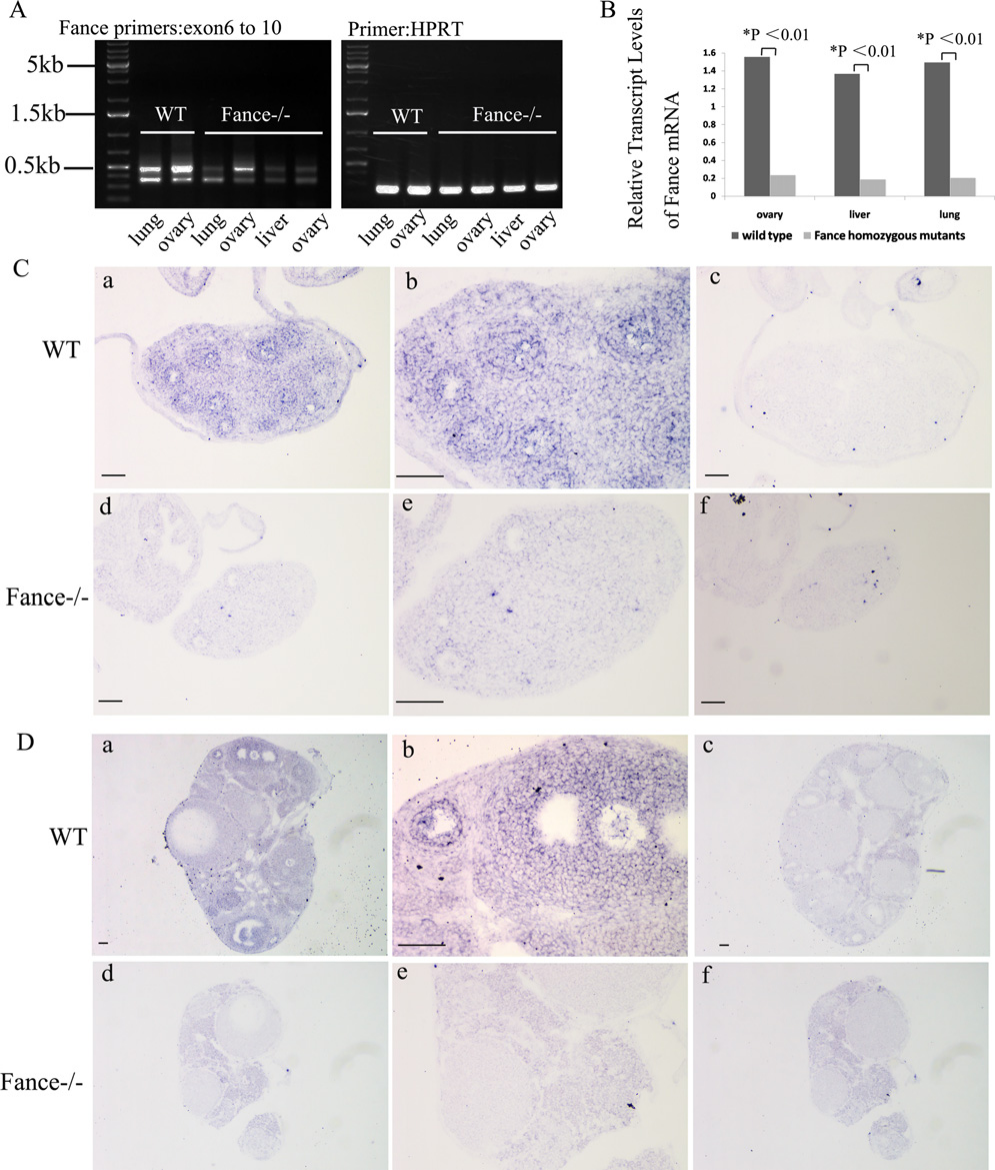
*Fance* mRNA is altered in *Fance*^-/-^female mice. (A) RT-PCR analysis of *Fance* transcripts. RT-PCR for Hprt was used as a control for RNA condition and concentration. (B) Relative transcript levels of *Fance* mRNA in *Fance*^-/-^ovaries, liver and lung compared with those in *Fance*^+/+^ mice, assayed by real-time RT-PCR. (C, D) In situ hybridization (ISH) assays for *Fance* expression in 2-week-old (C) and 8-week-old mouse ovary (D). *Fance* mRNA expression was detected in *Fance*^**+/+**^ ovary sections (a and b using antisense probe; c using sense probe); *Fance* mRNA was not detected above background in the *Fance*^-/-^ ovaries (d and e using antisense probe; f using sense probe). All Scale bars = 100μm.

The *Fance* gene has 10 exons and maps to mouse chromosome 17. The integration site of the lentivirus in family OVE2364E-2a2 was mapped tointron 8 of *Fance* (Fig 1A) using inverse PCR (see S1 Fig for the actual sequences). The lentiviral vector contains a tyrosinase minigene to allow visual genotyping. Mice with different coat colors have different genotypes (Fig 1B). Wild type mice (*Fance*^+/+^, non-transgenic) are white (albino), heterozygous mice (*Fance*^+/-^) are light gray and homozygous mutant mice (*Fance*^-/-^) are darker gray. PCR amplifications were done with primers that flank the lentiviral integration site to confirm the visual genotyping (Fig 1C). *Fance* expression levels were first assessed by RT-PCR using primers amplifying exons 6–10. Due to alternative splicing, the RT-PCR amplifications produced two bands in every sample (Fig 2A). DNA sequencing showed the top band contained exons 6–10 and the bottom band contained exons 6–8, and 10 in the tissues from wild type mice (data not shown). In the mutant tissues, neither band contains a properly spliced exon 9, so neither transcript encodes a wild-type Fance protein (data not shown).

Real time RT-PCR was used to quantitate the changes in transcript levels (Fig 2B). Relative transcript levels of *Fance* mRNA declined approximately 8-fold in homozygous mouse ovaries, liver and lung compared with those in wild type mice. Using in situ hybridization, *Fance* mRNA expression was detected in oocytes, granulosa cells and stromal cells of *Fance*^+/+^ mouse ovary, but was not detected above background in the *Fance*^**-/-**^ovary (Fig 2C and 2D).

### 2. Phenotypic characterization of *Fance*^-/-^mice

**Fig 3:**
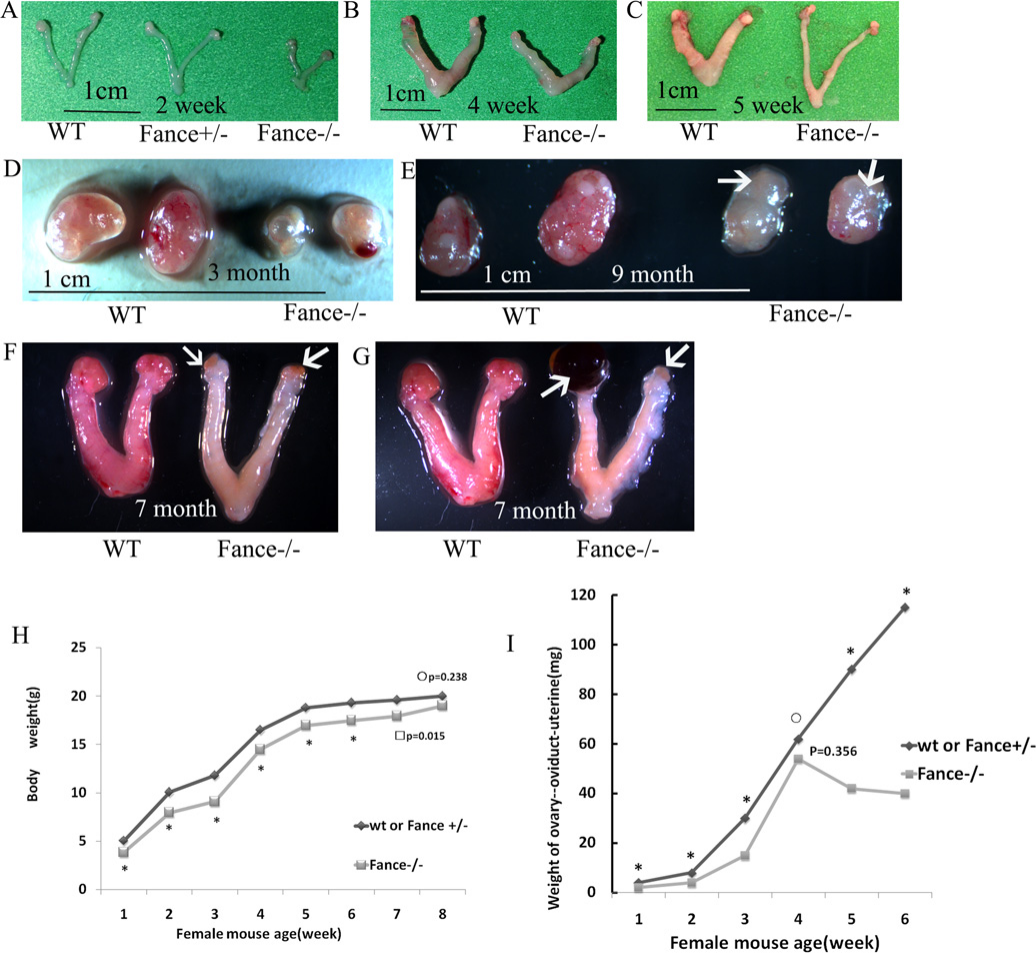
*Fance*^-/-^ reproductive tissues. (A-G) The development of the reproductive system (ovary+oviduct+uterus) in homozygous mutant and control mice. Older mutant ovaries show evidence of dysplasia (white arrows). (H) Growth curves. (I) Growth of the reproductive system.

Adult *Fance*^-/-^female mice looked normal and had no obvious morphological changes. Visceral organs showed no obvious abnormalities. However, ovarian sizes were significantly reduced in older homozygous mutant mice (Fig 3A–3G). No obvious follicles were seen on the surface of the mutant ovaries in mice that were 3 months of age or older. Intracystic hemorrhage and cysts were often seen in the mutant mice. The uterus of the *Fance*^-/-^mice became elongated with reduced blood circulation on the uterine surface (Fig 3A–3C, 3F and 3G). Ovarian tumors were not found in the *Fance*^-/-^ mice during the observation period (more than 1 year).

The body weight of *Fance*^-/-^ mice was lower than that of *Fance*^+/-^ or WT mice before 7 weeks of age (P<0.05, n = 8, Fig 3H). The weight of the reproductive system (ovary-oviduct-uterus) in *Fance*^-/-^ mice appeared to plateau around 4 weeks of age (Fig 3I).

Mating studies confirmed that *Fance* ^-/-^ female mice show impaired fertility (Table 1). The infertility rate was 50% (Table 1).The number of pregnancies and average litter sizes for *Fance*^-/-^ female mice were both significantly smaller than those in WT or *Fance*^+/-^ mice (P<0.001 and P = 0.0037, Table 1).

**Table 1:** Female fertility.

Genotype	*Fance*^-/-^	WT or *Fance*^+/-^
**Number**	12	6
**Number of pregnant mice**	6	6
**Times of pregnancy**	0.75±0.25	4.67 ± 0.21^a^
**Total number of kids**	44	214
**Living kids at weaning**	38	206
**Average litter size**	4.89±0.86	7.64±0.42^b^

a: P<0.0001 Times of pregnancy

b: P = 0.0037 Average litter size

### 3. Ovarian hypoplasia in *Fance*^-/-^ mice

We harvested ovaries from *Fance*^+/+^ and *Fance*^-/-^ mice at 6 different age points (5 days, 2weeks, 3weeks, 4weeks, 5weeks and 6weeks).Ovaries of mutant mice were smaller than those of WT at every age.

**Fig 4:**
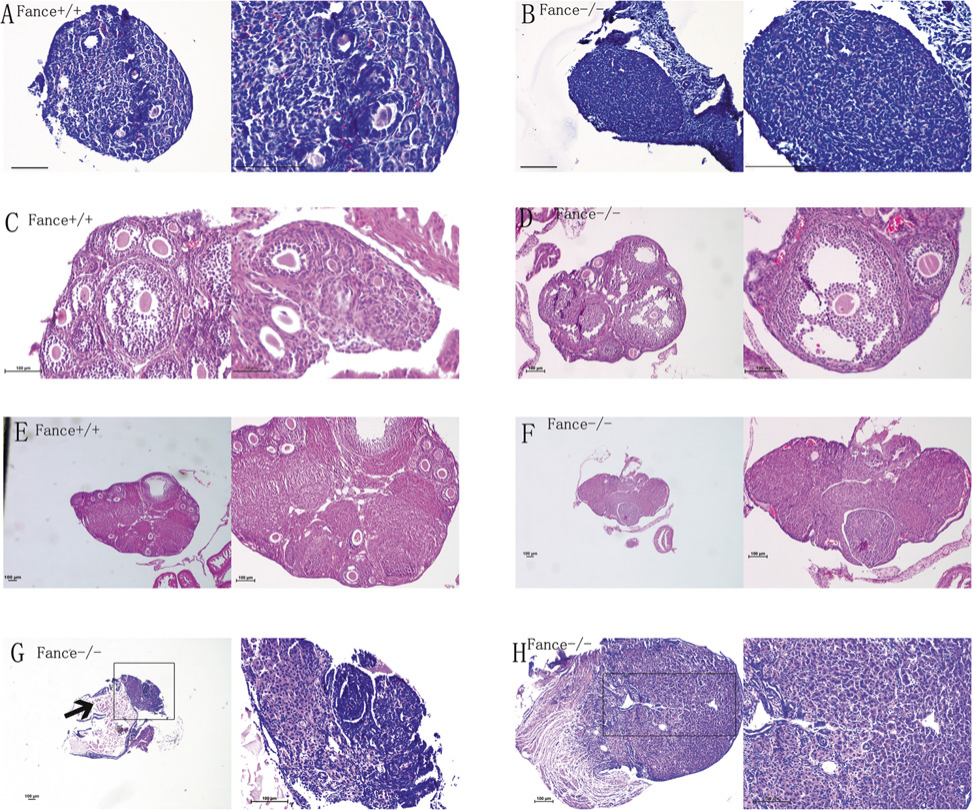
Histology sections of mouse ovaries at different ages (all bars = 100μm, except bar = 50μm in right side picture of C). (A), (B) ovary sections from5-day-old mice; (C), (D) ovary sections from3-week-oldmice; (E), (F) sections from6-week-oldovaries; (G), (H) sections from a 7-month-old mouse. A cyst containing fluid and bleeding was present in one *Fance*^-/-^ ovary, and adjacent cells (arrow in G) showed hyperplasia.

Ovaries were fixed, paraffin embedded, serially sectioned and sections were stained with H&E (Fig 4). Follicle numbers were quantitated at each age (see S2 Fig). The numbers of primordial and primary follicles in *Fance*^+/+^ mice of 5d age were 504.5±25.3 and 89.5±8.2, respectively. In contrast, the numbers of primordial and primary follicles in *Fance*^-/-^mutant mice of the same age were 14.5±2.6 and 9.5±2.6. The numbers of primordial, primary, secondary and antral follicles were respectively 169.7±17.9, 47.5±6.4, 39.2±3.8, 15.2±3.3 in 3- week-old *Fance*^+/+^ mice; they were 4.5±1.3, 3.7±1.0, 8.0±1.4 and 5.0±1.4 in *Fance* ^-/-^ mice of the same age(n = 4). Corpus luteums and interstitial cells were seen in *Fance* ^-/-^ovaries at 6 weeks of age, but no obvious follicles were seen in serial sections of the whole ovary (n = 4, Fig 4E and 4F). One ovary had a cyst in a 7-month-old *Fance* ^-/-^ mouse. The other ovary had no normal follicles (Fig 4G and 4H).

After superovulation, 44 one-cell embryos were collected from 2 *Fance*^+/+^ mice and 35 one-cell embryos were harvested from 2 *Fance*^+/-^ mice; however, only 4 one-cell embryos were collected from 2 *Fance*^-/-^ mice.

### 4. Changes of vaginal exfoliated cells in *Fance*^-/-^ mice

**Fig 5:**
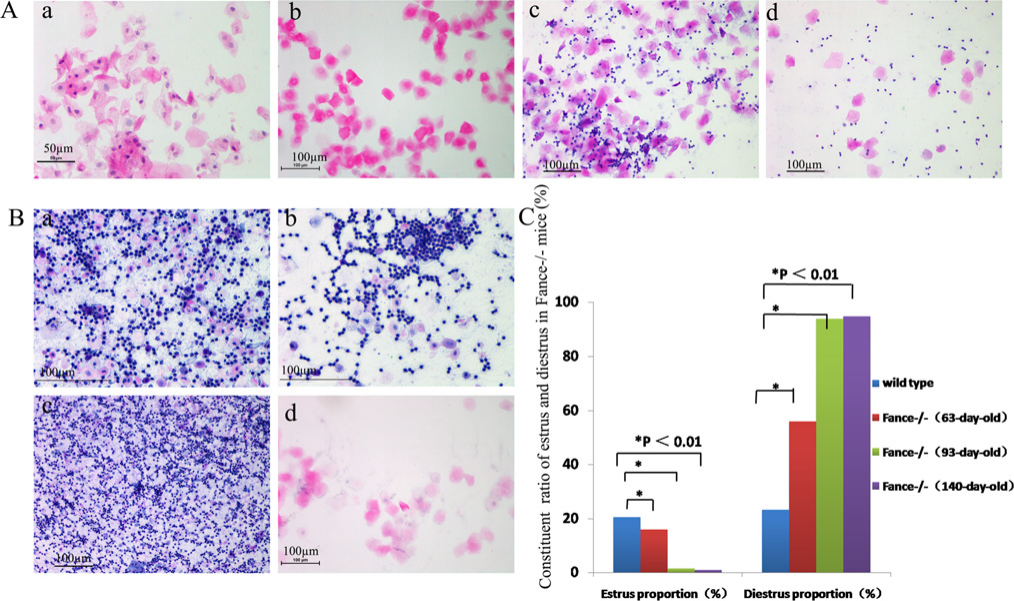
Vaginal exfoliated cells stained with HE. (A) WT mice at reproductive age showed the 4 stages of estrus: proestrus(a), estrus(b), metestrus(c) and diestrus(d). (B) *Fance*^-/-^ mice at 13 weeks of age had irregular estrous cycles. Diestrousphase (a,b,c) was almost always seen and estrus phase(d) was rarely seen. (C) Ratios of estrus in *Fance*^-/-^mice at three different ages were significantly lower than those in *Fance*^+/+^ mice.

*Fance*^+/+^ mice at three different ages (9 weeks, 13 weeks, and 20 weeks) showed a 4–5 day estrous cycle with four stages, proestrous, estrous, diestrous and metestrous (Fig 5Aa–5Ad). *Fance*^-/-^ mice all showed irregular estrous cycles at the same three ages. Estrous phases were seen in *Fance*^-/-^ mice at 9 weeks of age. However, diestrous was predominant in *Fance*^-/-^mice at 13 weeks (Fig 5B) and 20 weeks (Fig 5C).

### 5. Few apoptotic cells in *Fance*^-/-^ ovaries

**Fig 6:**
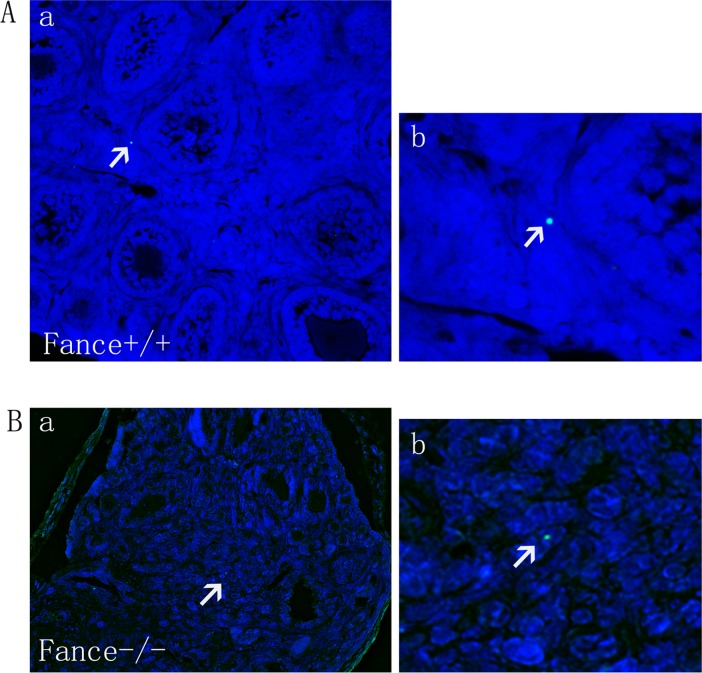
TUNEL assay. (A, B) TUNEL staining of mouse ovaries showed one apoptotic cell (green) in *Fance*^+/+^ (A) and *Fance*^-/-^ (B) sections (white arrows). The sections were counterstained with DAPI (blue).

2-week-old mice were assayed for apoptotic cells in ovaries (n = 3). Few apoptotic cells were found in the ovaries of either *Fance*^-/-^ or *Fance*^+/+^ mice. 1 or 2 apoptotic cells could be seen in cross sections of the ovary (Fig 6).

## Discussion

In the study, we describe a new mouse line with insertional inactivation of the *Fance* gene. The mice have a lentiviral transgene inserted into intron 8 of *Fance*. *Fance* RNA transcription is significantly reduced in the homozygous mutant mice, and none of the transcripts encode a wild-type version of the protein. The *Fance*^-/-^ mice show ovarian dysplasia and severely reduced numbers of follicles at all post-natal ages examined. The mutant females have impaired fertility and an aberrant estrous cycle. The ovarian phenotype of the *Fance*^-/-^ mice resembles that of women suffering from primary ovarian insufficiency (POI) characterized by a lack of developing follicles.

Our *Fance*^-/-^mouse is the first reported *Fance* mouse mutant. FANCE is an important protein that participates in DNA repair in response to interstrand crosslink damage [26]. Efficient and accurate DNA repair is likely to be essential to maintain genomic integrity in germ cells, thereby protecting the coding sequences of the germ line. At least 18 FA proteins (FANC-A, -B, -C, -D1, -D2, -E, -F, -G, -I, -J, -L, -M, -N, -O, -P, -Q,-S, and UBE2T) and 4 Fanconi-associated proteins (FAAPs; FAAP16, FAAP20, FAAP24, and FAAP100) are involved in the FA pathway [8, 9, 27]. FANCE is a component of the FA core complex (composed of 8 FA proteins and 2 FA-associated proteins), and activation of this complex controls the FA pathway. When DNA is damaged by ionizing radiation, ultraviolet radiation or replication errors, FANCE serves as a bridge between the FA core complex and FANCD2 through direct binding of FANCC and FANCD2 [28, 29].

Like previously described mouse mutants (*Fanca*^-/-^, *Fancc*^-/-^ and *Fancg*^-/-^), *Fance*^-/-^ female mice did not display morphological anomalies and showed no tumor formation in nearly one year of observation. Although there were some differences in phenotype, all the FA mutant female mice described to data, show gonadal dysgenesis and impaired fertility [10–20]. Ovarian tissues show depletion of follicles and interstitial cell hyperplasia in the FA mutant mice.

In our study, *Fance* deficient mice were found to have a similar severe lack of primordial and primary follicles by 5 days after birth. In other words, the ovarian reserve is significantly reduced in *Fance* deficient mice. Other FA mutant mice have a serious lack of primordial germ cells (PGCs), as early as 11.5dpc and 12.5dpc respectively in *Fanca* and *Fancc* deficient mice [10,30]. The PGC number at 8.5dpc was similar between *Fanca*^-/-^ mice and control mice; however, the PGC number of mutant mice was only 50% of control mice by11.5dpc. This suggests that the *Fanca* gene plays an essential role in PGC proliferation. In *Fancm* mutant mice, the proliferation of PGCs (11.5dpc to 13.5dpc) was decreased and apoptosis was not increased [17]. We predict that *Fance* is similarly essential for proliferation of PGCs in early embryos.

Women are born with a finite pool of primordial follicles. Less than 1% of the follicles become mature and ovulate during the years before menopause. The vast majority of primordial follicles undergo atresia [31]. There is clinical evidence for POI in FA patients. For instance, the menopause year of FA patients is usually before the age of 40 and female patients often show decreased reproductive function (nearly 50% infertility) [32]. In addition, a study from South Korea has shown that polymorphisms within the *Fanca* gene increase the risk of women suffering from premature ovarian failure [33]. Based on the genetic studies in mice, it seems likely that FA females will typically have a reduced ovarian reserve. Although many FA genes have been identified and studied, it is still unclear what role FA proteins play in the regulation of primordial germ cell proliferation. Since there are still unsolved mysteries for FA, we hope that the new *Fance* mouse will be a valuable model for POI.

## Conclusion

Ovarian dysplasia, severe lack of follicles and impaired fertility are seen in *Fance*^-/-^female mice.

## Supporting Information

S1 Fig DNA sequencing of the integration site. The junction sequences map to intron 8 of *Fance*.
(TIF)

S2 Fig Follicle numbers at two different ages. (A,B) follicle numbers in 3-week-old ovaries;(C,D) follicle numbers in 5-day-old ovaries.
(TIF)
