# Supplementary figures and images for: Primary Ovarian Insufficiency Induced by Fanconi Anemia E Mutation in a Mouse Model
Source: PLoS One. 2016 Mar 3;11(3):e0144285. doi: 10.1371/journal.pone.0144285 (PMC4777492; doi:10.1371/journal.pone.0144285)

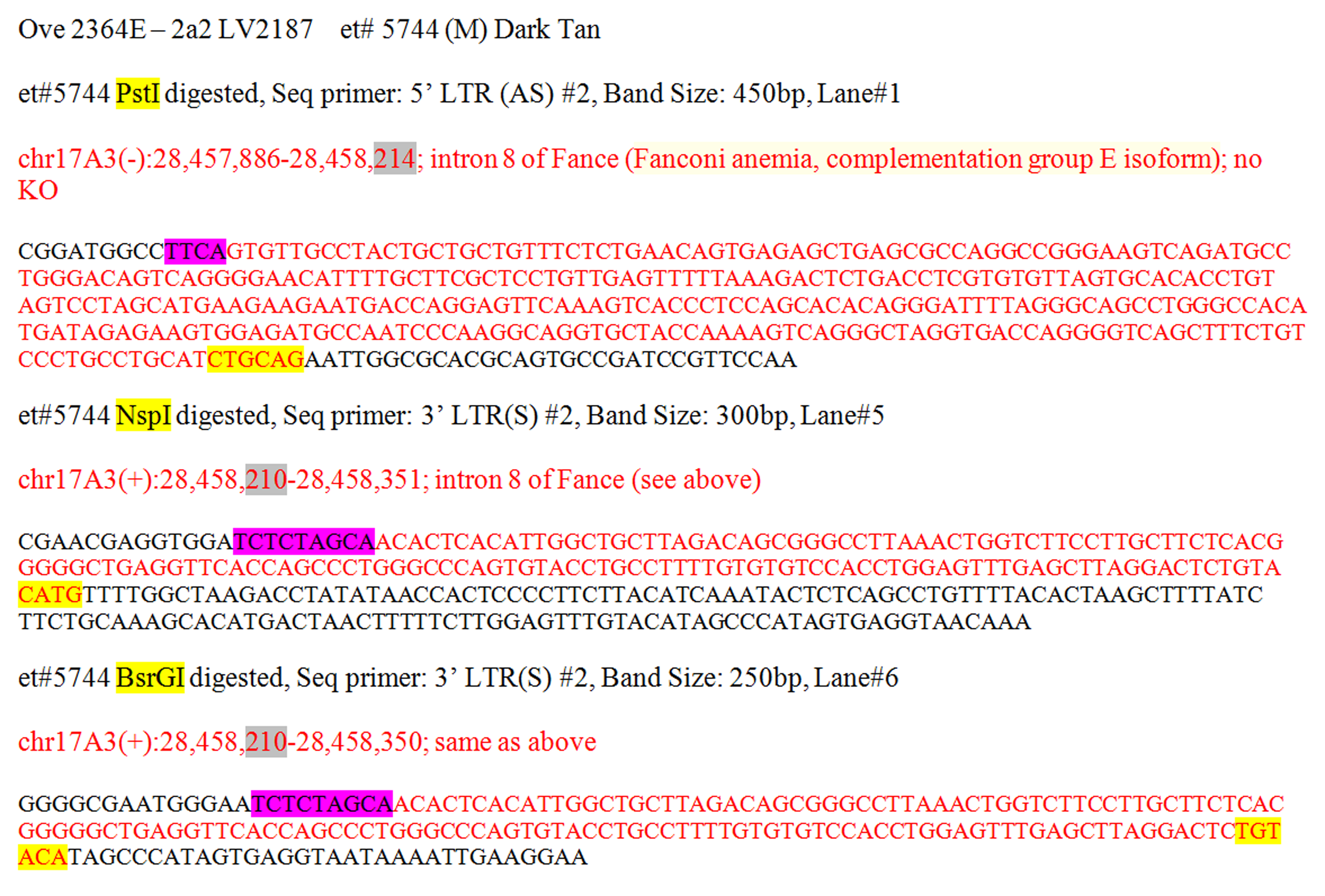

Supplement: S1 Fig — The junction sequences map to intron 8 of Fance. (TIF) [file pone.0144285.s001.tif]

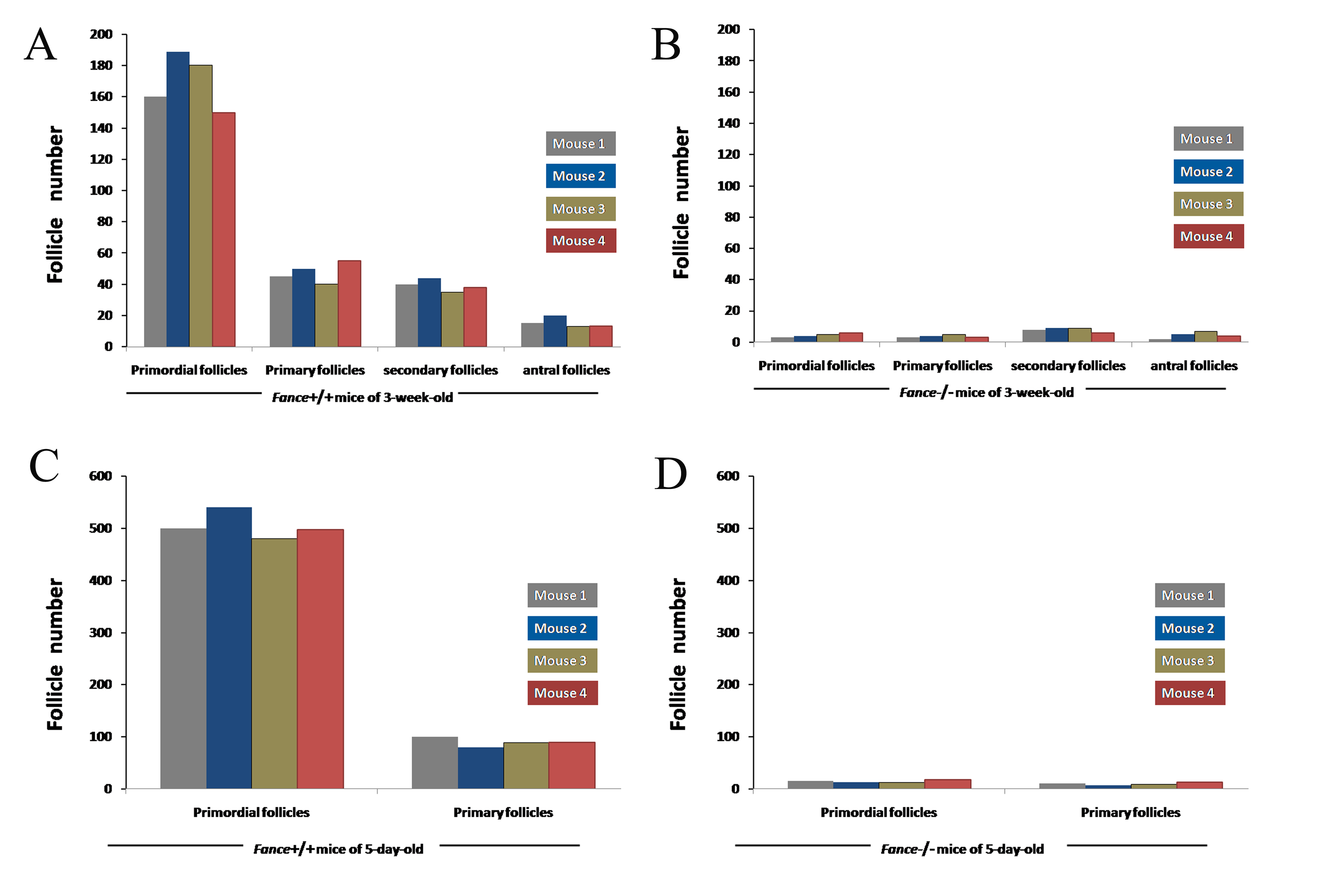

Supplement: S2 Fig — (A,B) follicle numbers in 3-week-old ovaries;(C,D) follicle numbers in 5-day-old ovaries. (TIF) [file pone.0144285.s002.tif]
